# Transcriptome Analysis of the Cecal Tonsil of Jingxing Yellow Chickens Revealed the Mechanism of Differential Resistance to *Salmonella*

**DOI:** 10.3390/genes10120979

**Published:** 2019-11-28

**Authors:** Fei Wang, Jin Zhang, Bo Zhu, Jie Wang, Qiao Wang, Maiqing Zheng, Jie Wen, Qinghe Li, Guiping Zhao

**Affiliations:** 1 Institute of Animal Sciences, Chinese Academy of Agricultural Sciences, Beijing 100193, China; feiwang2840@163.com (F.W.); zhangjin0913@126.com (J.Z.); boyzhubo@126.com (B.Z.); wangjie4007@126.com (J.W.); wangqiao@caas.cn (Q.W.); zhengmaiqing@caas.cn (M.Z.); wenjie@caas.cn (J.W.);; 2 State Key Laboratory of Animal Nutrition, Beijing 100193, China; 3 School of Life Science and Engineering, Foshan University, Foshan 528000, China

**Keywords:** cecal tonsil, transcriptome, *Salmonella*, chicken, susceptibility

## Abstract

*Salmonella* is one of the most common food-borne pathogens. It can be transmitted between chickens, as well as to people by contaminated poultry products. In our study, we distinguished chickens with different resistances mainly based on bacterial loads. We compared the cecal tonsil transcriptomes between the susceptible and resistant chickens after *Salmonella* infection, aiming to identify the crucial genes participating in the antibacterial activity in the cecal tonsil. A total of 3214 differentially expressed genes (DEGs), including 2092 upregulated and 1122 downregulated genes, were identified between the two groups (fold change ≥ 2.0, padj < 0.05). Many DEGs were mainly involved in the regulation of two biological processes: crosstalk between the cecal tonsil epithelium and pathogenic bacteria, such as focal adhesion, extracellular-matrix–receptor interaction, and regulation of the actin cytoskeleton and host immune response including the cytokine–receptor interaction. In particular, the challenged resistant birds exhibited strong activation of the intestinal immune network for IgA production, which perhaps contributed to the resistance to *Salmonella* infection. These findings give insight into the mRNA profile of the cecal tonsil between the two groups after initial *Salmonella* stimulation, which may extend the known complexity of molecular mechanisms in chicken immune response to *Salmonella*.

## 1. Introduction

*Salmonella* is an important cause of food-borne and zoonotic disease, which can colonize in chickens. It also poses a serious threat to people’s health via the consumption of contaminated meat and eggs [1]. Many measures have been taken to reduce salmonellosis during poultry production, including the improvement of the breeding environment, sterilization and vaccination [2]. However, cases of salmonellosis happen occasionally [3]. Therefore, new efficient and permanent strategies are necessary to control this disease. These strategies require an understanding of the interaction between chickens and bacteria.

Chickens are normally infected by *Salmonella* via the fecal–oral route. In young chickens, the innate immune mechanism is particularly crucial during infection with *Salmonella*, because of the immature adaptive immunity [4]. The intestinal epithelium is the first physical and immunological barrier against enteric pathogens, because the epithelial cell layer can reduce invasion and localization of *Salmonella* to the liver and spleen tissues [5]. Besides the physical barrier, Toll-like receptors (TLRs) on the cell membrane, especially TLR4, play an essential role in the recognition and defense against *Salmonella* infection [6]. The activation of the TLR signal pathway induces the secretion of pro-inflammatory cytokines to control bacterial growth [7]. Previous research has shown that the expression of genes related to the TLR4-MyD88-dependent pathway slightly increased on the first and third day after *Salmonella* infection in the caecum [8]. In addition to cytokines, IgA and IgG, secreted by the lymphocytes of the mucosa, participate in the host’s intestinal immune response, and the concentrations increased after the *Salmonella* invasion [9,10,11,12]. During bacterial–epithelial crosstalk, a series of changes occur in the gut, including intestinal flora and metabolism. *Salmonella* relies on the virulence factors, T3SS-1 and T3SS-2, to trigger intestinal inflammation in adult mice [13]. During invasion, *Salmonella* reduces the intestinal concentration of butyrate by the depletion of Clostridium [14]. Inflammation and butyrate decreases transform the metabolism of epithelial cells from mitochondrial β-oxidation of fatty acids to anaerobic glycolysis [15]. Gut morphology, such as villous height and crypt depth, can be affected by *Salmonella* stimulation, because *Salmonella* could lead to apoptosis of cells [9].

The cecal tonsil is a crucial gut-associated lymphoid tissue and plays a major role in controlling the entry of bacteria and other pathogens into the cecum [16]. Many studies have focused on the splenic transcriptome after infection with *Salmonella* [17,18] to reveal the host’s immune-related genes and pathways, because the spleen primarily participates in the recognition and clearance of bacteria. In their research, cytokine–cytokine receptor interaction pathways were significantly enriched after the *Salmonella* challenge. The genes involved in Forkhead box O (FoxO) and mitogen-activated protein kinase (MAPK) signaling pathways were identified as the potential markers related to host resistance against *Salmonella*. So far, little is known about the cecal tonsil transcriptome change after infection with *Salmonella*, especially between resistant and susceptible chickens. In this study, we compared the mRNA expression of the cecal tonsils between resistant and susceptible chickens to elucidate effective mechanisms of host resistance against *Salmonella*.

## 2. Materials and Methods

### 2.1. Ethics Statement

Ethical approval on animal care and experimental procedures were performed in accordance with the Animal Ethics Committee of the Institute of Animal Sciences, Chinese Academy of Agricultural Sciences (IAS-CAAS, Beijing, China).

### 2.2. Animals and Experimental Design

The *Salmonella* Typhimurium (ST, 21484 standard strain) was purchased from China Industrial Microbial Culture Preservation Center (Beijing, China). The bacteria were resuscitated overnight in Luria–Bertani (LB) broth (Amresco, Washington, DC, USA) at 37 °C in an orbital shaking incubator at 150 rpm. After recovery, ST was cultured for 12 h, and concentrated in a centrifuge. The final number of colony forming units (CFU) was determined by plating serial dilutions. Jingxing Yellow Chickens were obtained from the Changping Experimental Base of Institute of Animal Sciences (Beijing, China). All the chickens were checked for the presence of *Salmonella* by culturing faecal samples in buffered peptone water overnight with shaking at 150 rpm and spreading the samples on brilliant green agar (37 °C, 18–24 h) [8]. According to the results, the positive chickens were eliminated. A total of 146 1-day-old chicks were raised in separate cages at the experimental center of China Agricultural University (Beijing, China) with free access to feed and water. At 7 days of age, the chicks were orally inoculated with 1 mL culture containing 2.5 × 10^10^ CFU *Salmonella* Typhimurium. The birds’ blood samples, livers and cecal tonsils were collected at 3 days post infection, respectively. The cecal tonsils were collected and placed in an −80 °C freezer for short-term storage, the blood was allowed to clot and the serum was stored, and the livers were used for the later measurement of bacterial loads.

The lysozyme concentration in the serum was measured by Chicken lysozyme (LZM) ELISA Kit (Cusabio, Wuhan, China) [19]. In brief, standards and serum diluent with biotin-conjugated lysozyme were pipetted into the wells, where the lysozyme antibody had been pre-coated. After incubation, the wells were washed and avidin conjugated Horseradish Peroxidase (HRP) was added to the wells. Following incubation and wash, a substrate solution was added to the wells, and color developed. The intensity of the color was measured by a microplate reader. According to the standards, the lysozyme in the serum was calculated. For the determination of bacterial loads, we first took tissues of the same weight, then ground and diluted these tissues to different concentrations, and finally plated the same amount of serial dilutions onto a MacConkey agar medium (Thermo Fisher Scientific, Waltham, MA, USA) to determine the presence of ST and calculate the bacterial loads. Finally, according to the liver bacterial loads and clinical signs including diarrhea, drooping wings and dying, 14 chicks were identified as susceptible (severe clinical signs and liver loads >10^4^ CFU) or resistant (slight clinical sighs and liver loads <10^4^ CFU) birds, from which the cecal tonsils were selected for RNA sequencing (RNA-seq).

### 2.3. Total RNA Isolation, cDNA Library Construction, and Sequencing

The cecal tonsil RNA was extracted from the 14 chickens using a QIAGEN kit (Qiagen, Hilden, Germany). The quality and quantity of the total RNA were assessed by a 2100 Bioanalyzer and RNA 6000 Nano kit (Agilent, Santa Clara, CA, USA). For the mRNA library construction and deep sequencing, 3 µg total of RNA was prepared using the TruSeq RNA Sample Preparation Kit (Illumina, San Diego, CA, USA) to capture the coding transcriptome. After purification, the RNA was fragmented using divalent cations at 95 °C. The cleaved RNA fragments were reversely transcribed into first-strand cDNA using TruSeq RNA Library Preparation Kit, followed by second-strand cDNA synthesis. After cDNA fragments purification and adaptor ligation, sequencing was performed on the HiSeq X Ten platform (Illumina, San Diego, CA, USA).

The sequence data reported in this paper was deposited in the Genome Sequence Archive of BIG Data Center, Beijing Institute of Genomics (BIG), Chinese Academy of Sciences and are publicly accessible at http://bigd.big.ac.cn/gsa/s/ZzF2353M (accession number: CRA002119).

### 2.4. Analysis of Differentially Expressed Genes (DEGs) and Pathway Enrichment

The quality control of reads was analyzed by FastQC software (v0.10.1) [20]. Sequence adapters and low-quality reads were eliminated. The clean reads were mapped to the chicken reference genome (Gallus gallus 5.0) by Hisat2 [21]. The expression quantities of the mapped transcripts were calculated using Htseq [22]. Analysis of the DEGs were conducted by DESeq2 [23]. Genes with fold-change ≥2 and padj <0.5 were considered to be DEGs. To assess the variation between samples, principal component analysis (PCA) was conducted by gmodels (based on the gene transcripts) (https://CRAN.R-project.org/package=gmodels) and hierarchical clustering (based on DEGs) was conducted by pheatmap (https://CRAN.R-project.org/package=pheatmap), respectively. Volcano plots were performed by ggplot2 to provide an overview of the DEGs [24]. Based on the DEGs, the gene enrichment analysis was conducted by Gene Ontology (GO) functional enrichment analysis [25] and KOBAS [26].

### 2.5. Quantitative Real-time PCR

After reverse transcription, qPCR was carried out using a SYBR Fast qPCR Master Mix (KAPA, Wilmington, MA, USA). The qPCR amplification system was as follows: 3 µL of cDNA (10-fold dilution), 5 µL of 2× SYBR Master Mix, 0.2 µL of ROX, 0.25 µL of each primer, and adding water to 10 µL. The samples were amplified using the real-time PCR Detection System ABI 7500 (Applied Biosystems, Foster City, CA, USA). The qPCR cycle parameters were as follows: 95 °C for 3 min, 40 cycles of 95 °C for 3 s, and 60 °C for 34 s. Three independent replicates were used for each assay. The 2^−ΔΔ*C*T^ method was used to calculate the relative abundance of transcripts [27]. The correlation analysis was conducted between the relative expression calculated by the q-PCR and the fold-change by RNA-seq.

## 3. Results

### 3.1. Overview of the Immune Response between Susceptible and Resistant Chickens

**Figure 1:**
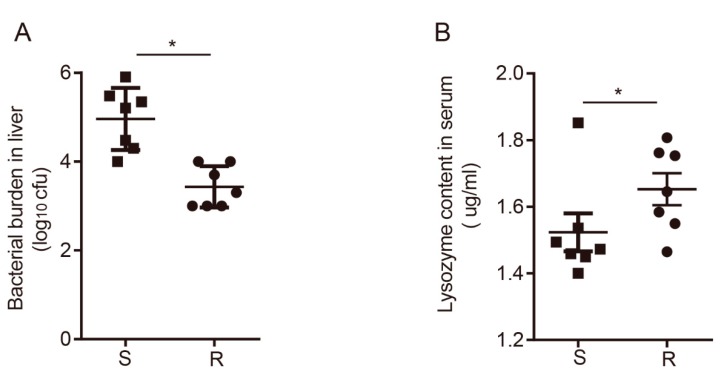
Bacterial burden and lysozyme content in chickens. (**A**) The liver was plated onto the MacConkey agar medium and the bacterial burden was determined by counting. (**B**) Lysozyme concentration in the serum was measured by ELISA. Data are shown as means ± SD. Data with asterisks were statistically significant (*p* ≤ 0.05).

To choose that candidate individuals of the chickens for RNA-seq, the liver bacterial load was measured by counting the number of colonies on the MacConkey agar. In addition, the lysozyme content in the serum was evaluated by ELISA. Seven individuals were selected in combination with the clinical symptoms in each group. The bacterial loads of the resistant chickens were significantly lower than those in the susceptible chickens, as seen in Figure 1A. However, as seen in Figure 1B, the lysozyme contents of the resistant chickens were higher than those of the susceptible chickens, which indicated that the resistant chickens cleared ST effectively.

### 3.2. Sequencing of Cecal Tonsil Transcriptomes

RNA-seq of the cecal tonsils yielded >40 Mb in the 14 samples. Around 93% of the clean reads had quality scores exceeding the Q30 value. The data demonstrated the reliability of the RNA-seq and could be used for data analysis. After eliminating the interference reads, the clean reads accounted for >93%, as shown in Supplementary Table S1. A total of 17,296 genes were detected, and 4864 genes were novel.

### 3.3. Differentially Expressed mRNAs Responding to ST Infection in Cecal Tonsil

**Figure 2:**
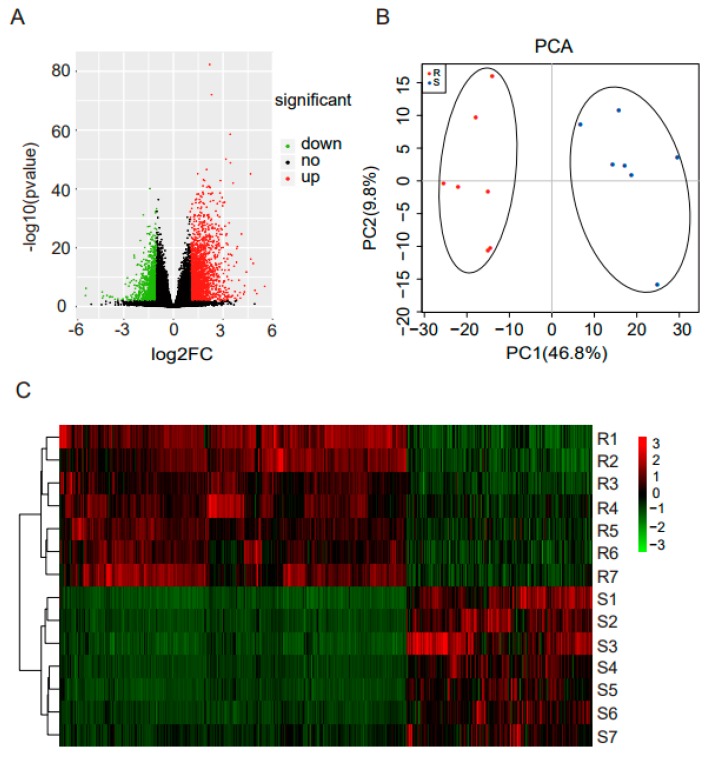
RNA-seq analysis and identification of genes differentially expressed between susceptible and resistant chickens. (**A**) Volcano plot showing DEGs. The red dots represent significantly upregulated genes (log2 FC ≥1 and padj <0.05); green dots represent significantly downregulated genes; and black dots represent genes with no significant change. (**B**) PCA of the RNA-seq data based on all the identified genes. The susceptible and resistant chickens were distinctly clustered. (**C**) The heat map of the RNA-seq data based on DEGs. S, susceptible chickens; R, resistant chickens.

The differentially expressed mRNAs between susceptible and resistant chickens were identified using DESeq2. As seen in Figure 2A, there were 3214 genes significantly differentially expressed after ST stimulation. As shown in Supplementary Table S2, 2092 genes were upregulated and 1122 were downregulated. PCA indicated that two groups of birds were distinctly clustered, as shown in Figure 2B. As seen in Figure 2C, hierarchical clustering (based on all DEGs) was consistent with PCA, and showed that there were more upregulated than downregulated genes.

### 3.4. Quantitative Real-Time PCR Validation

**Figure 3:**
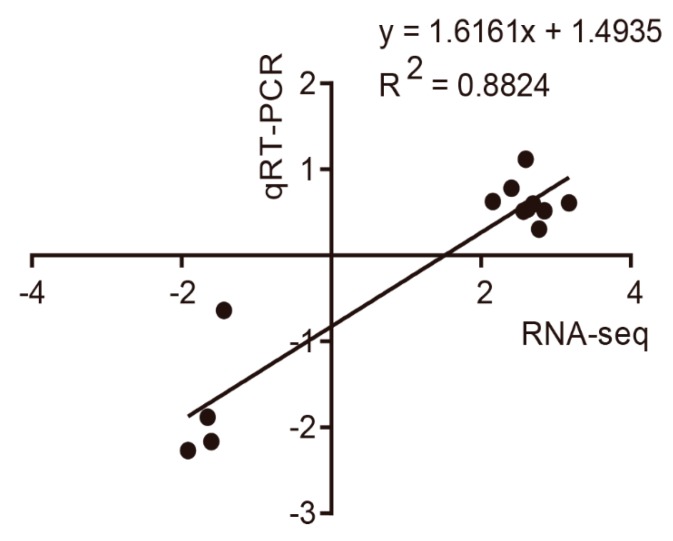
Correlation analysis of the relative expression levels of 13 DEGs between the RNA-seq and qPCR.

To verify the accuracy of the sequencing, qPCR was carried out. A total of 13 DEGs were randomly selected from RNA-seq. The specific primers shown in Supplementary Table S3 were designed using Oligo 6.0 software, and were subsequently synthesized by BGI (Beijing, China). As seen in Figure 3, the correlation coefficient between RNA-seq and qPCR was 0.8824, which indicated that the RNA-seq data was reliable for subsequent analysis.

### 3.5. Functional Enrichment Analysis of the DEGs

**Figure 4:**
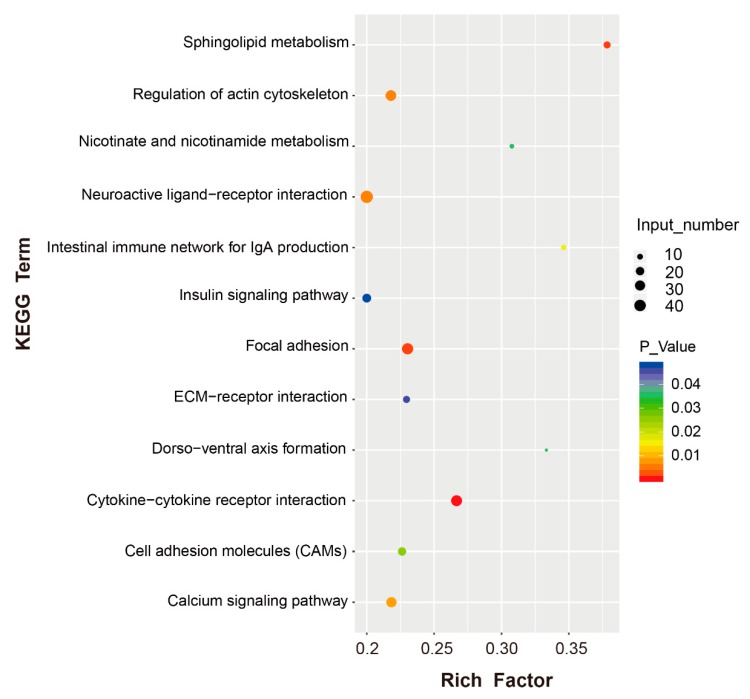
KEGG pathway analysis of DEGs. The y axis represents the significantly enriched KEGG terms based on the DEGs. The x axis represents rich factors (rich factor = number of DEGs enriched in each term/number of all genes in each term). Color represents significance, and size of the bubble represents the number of DEG enriched in the pathway (*p*-Value: before correction).

Potential function analysis of all the DEGs was performed using GO and KEGG enrichment. Some significantly enriched GO terms were mainly involved in cell communication and adhesion, integral components of membranes, response to stimulus and the immune system, as shown in Table 1. After KEGG pathway analysis, 20 pathways were significantly enriched, as seen in Figure 4 and Supplementary Table S4. Several of these pathways were related to metabolism, including sphingolipid, nicotinate and nicotinamide metabolism. Many of the pathways were involved in the immune response, including the intestinal immune network for IgA production and the cytokine–receptor interaction. In addition, four enriched pathways were related to the interaction and adhesion between intestinal microorganisms and epithelial cells, including focal adhesion, regulation of actin cytoskeleton, cell adhesion molecules (CAMs) and the extracellular matrix (ECM)–receptor interaction. The DEGs relating to the cytokine–receptor interaction and focal adhesion were examined further. The relative DEGs of each pathway are shown in the Supplementary Tables S5 and S6.

## 4. Discussion

An increasing number of studies focus on the transcriptome changes in chickens after *Salmonella* stimulation, especially the spleen transcriptome. However, there are few studies on the changes in the cecal tonsil transcriptome. In order to understand the function and transcriptome changes in the cecal tonsil after *Salmonella* infection, we compared the cecal tonsil expression profiles between susceptible and resistant chickens. Apart from clinical symptoms, the bacterial burden in the livers and the lysozyme content in the serum were taken into consideration to evaluate the susceptibility and resistance of the chickens. The cecal tonsil expression profile was first detected by RNA-seq. A total of 3217 DEGs were identified, some of which were related to IgA production including AICDA, TNFRSF13C, CD86, TNFSF13B, CD80, ICOS, CCR9 and CD28. The significantly changed pathways related to the interaction between the bacteria and the epithelia and the immune response were identified in the cecal tonsil.

Many significantly enriched pathways, including focal adhesion, sphingolipid metabolism, regulation of actin cytoskeleton, CAMs and the ECM–receptor interaction were related to the bacterial–epithelial crosstalk, which occurs during the bacterial invasion of the intestine. In this process, a necessary step is the adhesion of bacterial pathogens to the intestinal epithelium, which is the basis for successful colonization and ultimate production of disease [28]. Recent studies suggest that adhesion relies on glycoconjugates induced by indigenous intestinal microflora on the host epithelial cells [29]. During this time, type III secretion, which *Salmonella* possesses [30], mediates the transfection of the bacterial proteins into the host cell membrane [31]. These secreted soluble molecules activate epithelial cells by a series of signaling pathways including Ca^2+^ transportation, resulting in actin cytoskeleton rearrangement and the entry of the bacteria into the cells. Sphingolipids, produced by both the host and specific bacteria, are closely related to metabolic and inflammatory pathways in host cells. The cellular levels and distribution of sphingolipids differ between inflamed and noninflamed intestinal tissue. Sphingolipids from a symbiotic microbe regulate homeostasis of host intestinal natural killer T cells, resulting in inflammatory bowel disease [32,33].

**Figure 5:**
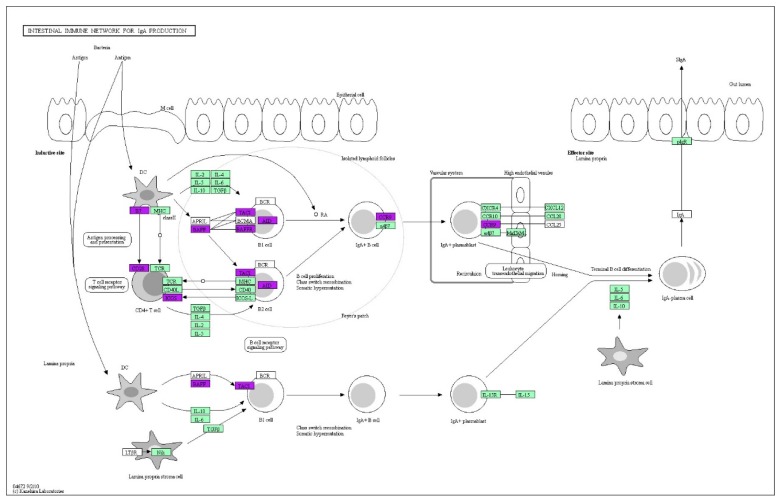
IgA production signaling pathway. The DEGs identified in this research are highlighted in purple.

Correspondingly, the host has specialized strategies to resist such invasion. In our research, the intestinal immune network for IgA production was enriched. IgA is the predominant antibody isotype produced at mucosal surfaces, and plays a critical role in the intestinal immune response and the prevention of tissue damage in inflammation [34,35,36]. When the invasion of pathogenic microorganisms is recognized by the intestinal immune system, IgA is induced and transported into the lumen [37]. It has been confirmed that pathogen-binding IgA regulates bacterial motility and protects the host from the pathogens [38]. Furthermore, IgA can also influence the bacterial invasion of epithelial cells by inhibiting the type III secretion system [39]. In our research, the genes crucial for IgA production are more highly expressed in resistant chickens, as shown in Figure 5, which means that the IgA signaling pathway was more active in this group.

Li et al. characterized the splenic transcriptomes of susceptible and resistant chickens [18]. Compared with his results, we found that 58 DEGs were the same, some of which were mainly involved in metabolism, as shown in Supplementary Table S7. Metabolism is essential for the host to produce an effective immune response against pathogenic microorganisms. Previous research has revealed that during the recognition of microbial ligands, macrophages initiate upregulation of glycolysis. The energy of glycolysis supports antimicrobial inflammation and secretion of cytokines [40,41]. It has been shown that a glucose-rich diet improves host survival rate in systemic fungal infection [42]. The spleen and the cecal tonsil are two critical immune organs that may have metabolic differences between susceptible and resistant chickens after *Salmonella* infection.

## 5. Conclusions

Our research was aimed at analyzing the transcriptome of cecal tonsils of susceptible and resistant chickens after *Salmonella* infection. A total of 3214 DEGs were identified between them. DEGs were mainly involved in two biological processes, the interaction and crosstalk between the cecal tonsil epithelium and the host immune response, and the immune response of the host. Importantly, the adhesion between *Salmonella* and the intestine is a crucial signaling pathway, which plays an important role in the gut pathological changes after *Salmonella* infection. In addition, there was stronger activation of the intestinal immune network for the IgA production signaling pathway in resistant chickens, which may contribute to the protection of chicken from *Salmonella* invasion.

## Figures and Tables

**Table 1:** Significant enriched biological processes by gene ontology analysis based on the DEGs.

Term	Description	Count	Corrected *p*-Value
GO:0023052	signaling	237	0.002
GO:0007154	cell communication	237	0.002
GO:0050896	response to stimulus	304	0.002
GO:0007165	signal transduction	219	0.003
GO:0002376	immune system process	98	0.004
GO:0007155	cell adhesion	72	0.007
GO:0022610	biological adhesion	72	0.007
GO:0016020	membrane	355	0.007
GO:0016021	Integral component of membrane	236	0.009
GO:0044425	membrane part	276	0.009
GO:0098602	single organism cell adhesion	46	0.100
GO:0031224	intrinsic component of membrane	238	0.012
GO:0016337	single organismal cell–cell adhesion	43	0.023
GO:0051716	cellular response to stimulus	248	0.028
GO:0006955	immune response	52	0.032
GO:0005886	plasma membrane	165	0.032
GO:0071944	cell periphery	168	0.043
GO:0032501	multicellular organismal process	236	0.049

All DEGs between susceptible and resistant chickens were used to identify enriched biological functions (*p* < 0.05).

## Supplementary Materials

The following are available online at https://www.mdpi.com/2073-4425/10/12/979/s1, Table S1: Sample grouping and sequencing data quality control (XLSX), Table S2: The screened DEGs (FC ≥ 2; padi < 0.05) in this study (XLSX), Table S3: The specific primers for qRT-PCR in this study (XLSX), Table S4: The enriched pathways (XLSX), Table S5: The DEGs related to focal adhesion in this study (XLSX), Table S6: The DEGs related to cytokine-cytokine receptor interaction in this study (XLSX), Table S7: The same DEGs and some of which mainly involved in metabolism.
